# Total Variation with Overlapping Group Sparsity for Image Deblurring under Impulse Noise

**DOI:** 10.1371/journal.pone.0122562

**Published:** 2015-04-15

**Authors:** Gang Liu, Ting-Zhu Huang, Jun Liu, Xiao-Guang Lv

**Affiliations:** 1 School of Mathematical Sciences/Research Center for Image and Vision Computing, University of Electronic Science and Technology of China, Chengdu, Sichuan, P. R. China; 2 School of Science, Huaihai Institute of Technology, Lianyungang, Jiangsu, P. R. China; Nanjing University of Aeronautic and Astronautics, CHINA

## Abstract

The total variation (TV) regularization method is an effective method for image deblurring in preserving edges. However, the TV based solutions usually have some staircase effects. In order to alleviate the staircase effects, we propose a new model for restoring blurred images under impulse noise. The model consists of an ℓ_1_-fidelity term and a TV with overlapping group sparsity (OGS) regularization term. Moreover, we impose a box constraint to the proposed model for getting more accurate solutions. The solving algorithm for our model is under the framework of the alternating direction method of multipliers (ADMM). We use an inner loop which is nested inside the majorization minimization (MM) iteration for the subproblem of the proposed method. Compared with other TV-based methods, numerical results illustrate that the proposed method can significantly improve the restoration quality, both in terms of peak signal-to-noise ratio (PSNR) and relative error (ReE).

## Introduction

Image deblurring and denoising problems have been widely studied in the past decades. In the literature, it is widely assumed that observed images are the convolution of standard linear and space invariant blurring functions with true images plus some noise. Let *g* denote the blurred and noisy image, *h* the blur kernel, *f* the original image and *η* the noise. The image *f* is assumed to be a real function defined on a bounded and piecewise smooth open subset Γ of ℝ^2^. In general, the image formation process can be modeled as: *g* = *h* ⋆ *f* + *η*, where “⋆” denotes the two-dimensional convolution operation. Image deblurring is to estimate the true image *f* from the blurred and noisy image *g*. As is well known, image deblurring and denoising is a typically ill-posed problem [1, 2]. To handle this problem, regularization technique is usually considered to obtain a stable and accurate solution. In this way, we need to solve the following problem

$$\begin{array}{c} \min_{f}\psi \left(f\right)+\frac{\mu }{2}\int_{\Gamma }{\left|h⋆f-g\right|}^{2}dx,\; \end{array}$$ (1)

where the first term is called the regularization term, the second term is called the fidelity term (ℓ_2_-fidelity), *μ* > 0 is the regularization parameter, and *ψ* is the regularization functional.

Without loss of generality, a discretized image may have *n*_1_ × *n*_2_ pixels. Simply, in our work, we assume that *n*_1_ = *n*_2_ = *n*, then *f*, *g* and *η* are vectors of length *n* ^2^. It is easy to extend to any *n*_1_ × *n*_2_ image. Let *H* be the corresponding blurring matrix of *n* ^2^ × *n* ^2^ from *h* [3]. Then the discretized form of the minimization problem (1) is equivalent to the following matrix-vector form

$$\begin{array}{c} \min_{f}\psi \left(f\right)+\frac{\mu }{2}{∥Hf-g∥}_{2}^{2},\;\; \end{array}$$ (2)

where ‖ ⋅ ‖_2_ denotes the Euclidean ℓ_2_ norm. Notice that *H* is a matrix of block circulant with circulant blocks (BCCB) structure when periodic boundary conditions are applied [3].

How to choose a good regularization functional is an active area of research in the imaging science. In the early 1960s, D. L. Phillips [4] and A. N. Tikhonov [5] proposed the definition of *ψ* as an ℓ_2_-type norm (academically called Tikhonov regularization), that is, $\psi =\|Lf{\|}_{2}^{2}$ with *L* an identity operator or difference operator. Although the functional *ψ* of this type has the advantage of facilitating the calculations, it is rarely used in current practice because it has the drawback of penalizing discontinuities in resulting solutions, for instance, over-smoothing edges. Therefore, this is not a good choice since natural images have many edges.

To overcome this drawback, many different types of regularization functionals have been proposed. One popular model was introduced by Rudin, Osher and Fatemi (ROF) in [6]. They proposed a total variation (TV) regularization with an ℓ_2_-fidelity term (ℓ_2_-TV) for image restoration. Its corresponding minimization task is:

$$\begin{array}{c} \min_{f}{∥f∥}_{\text{TV}}+\frac{\mu }{2}{∥Hf-g∥}_{2}^{2},\;\;\mathrm{over}\;f\in \text{BV}\left(\Gamma \right), \end{array}$$ (3)

where BV(Γ) denotes the space of functions of bounded variation. ‖*f*‖_TV_ is defined by ${\left\|f\right\|}_{\text{TV}}≔\sum_{1⩽i,j⩽n}{\left\|{\left(\nabla f\right)}_{i,j}\right\|}_{2}=\sum_{1⩽i,j⩽n}\sqrt{{|{\left(\nabla_{x}f\right)}_{i,j}|}^{2}+{|{\left(\nabla_{y}f\right)}_{i,j}|}^{2}}$ which is called as isotropic TV, or ${\left\|f\right\|}_{\text{TV}}≔\sum_{1⩽i,j⩽n}{\left\|{\left(\nabla f\right)}_{i,j}\right\|}_{1}=\sum_{1⩽i,j⩽n}|{\left(\nabla_{x}f\right)}_{i,j}|+|{\left(\nabla_{y}f\right)}_{i,j}|$ which is named as anisotropic TV, where ‖ ⋅ ‖_1_ denotes the Euclidean ℓ_1_ norm. Operator ∇: ℝ^*n*^2^^ → ℝ^2×*n*^2^^ denotes the discrete gradient operator (under periodic boundary conditions) which is defined by (∇*f*)_*i*,*j*_ = ((∇_*x*_ *f*)_*i*,*j*_, (∇_*y*_ *f*)_*i*,*j*_), with

$$\begin{array}{c} {\left(\nabla_{x}f\right)}_{i,j}=\{\begin{array}{ccc} f_{i+1,j}-f_{i,j} & \mathrm{if} & i<n,\\ f_{1,j}-f_{n,j} & \mathrm{if} & i=n, \end{array} \end{array}$$

$$\begin{array}{c} {\left(\nabla_{y}f\right)}_{i,j}=\{\begin{array}{ccc} f_{i,j+1}-f_{i,j} & \mathrm{if} & j<n,\\ f_{i,1}-f_{i,n} & \mathrm{if} & j=n, \end{array} \end{array}$$

for *i*, *j* = 1, 2, ⋯, *n*, where *f*_*i*,*j*_ refers to the ((*j* − 1)*n* + *i*)th entry of the vector *f* (it is the (*i*, *j*)th pixel location of the *n* × *n* image, and this notation remains valid throughout the paper unless otherwise specified). More details for the definition of ‖*f*‖_TV_ can be referred to [7, 8].

Many methods have been proposed to solve the restoration model (3) such as the fast TV deconvolution (FTVd) [9, 10], the augmented Lagrangian method (ALM) [11–14], the dual methods [13, 15], and the split Bregman method [11, 16]. These studies provided many efficient iterative algorithms to make the computation on TV related models be more convenient for Gaussian noise removal. Furthermore, in many cases, the noise does not satisfy the Gaussian assumption, for instance, the noise may follow a Laplace distribution [17]. There has been a growing interest in using an ℓ_1_-fidelity term instead of the ℓ_2_-fidelity term for image restoration in many literature such as [8, 18–27] for considering another non-Gaussian noise–impulse noise. We consider the corresponding regularization model with an ℓ_1_-fidelity term as:

$$\begin{array}{c} {\min\psi \left(f\right)+\mu ∥Hf-g∥}_{1}. \end{array}$$ (4)

A classic approach is to use the TV regularizer by *ψ*(*f*) = ‖*f*‖_TV_, which we call ℓ_1_-TV. Recently, Yang *et al*. [20] used the FTVd method to solve the ℓ_1_-TV model fast. Guo *et al*. [8] proposed a fast algorithm for image restoration in the ℓ_1_-TV model. Their method was to add a penalty term by using the variable substitution method, which belongs to penalty methods in optimization. They employed an alternating minimization method to solve it. They also proved the convergence of their method. Numerical tests showed that their method got better restorations and faster than FTVd. Wu *et al*. [25] used ALM to solve the ℓ_1_-TV model. More recently, Chan *et al*. [26] proposed a constrained total variation (TV) regularization method for image restoration for ℓ_1_-TV. Their method used a box constrained projection to ensure the restored images stay in a given dynamic range. They used the alternating direction method of multipliers (ADMM) [11–14, 28–33] to solve the model based on augmented Lagrangian method. They got better results than other methods such as FTVd and ALM. Their numerical results showed that for some images where there are many pixels with values lying on the boundary of the dynamic range, the gain could get very high numerical superiority in the peak signal-to-noise ratio. As far as our knowledge goes, the box constrained projection in image restoration is significant both in practice and theory.

Although the TV regularization using in the restoration problems can recover sharp edges of a degraded image, it also gives rise to some undesired effects and transforms smooth signal into piecewise constants, the so-called staircase effects [34, 35]. To overcome this deficiency, one usual method is to replace the original TV norm by a high-order TV norm. The high-order TV regularization schemes have been studied so far mainly for overcoming the staircase effects while preserving the edges in the restored image. More details can be referred to [35–38]. The high-order TV based methods may have some other behaviors. For example, it may transform the smooth signal to over-smoothing and take more time to compute.

More recently, Bredies *et al*. [39] proposed total generalized variation for image restoration with overcoming the staircase effects while preserving the edges. Pryre and Fadili [40] considered group sparsity and overlapping group sparsity (OGS) for image processing, such as denoisng, compressed sensing. Their numerical experiments showed that the PSNR (defined in Section 5) gain by OGS is a consistent improvement on comparing with group sparsity across a wide range of natural images. Selesnick and Chen [41] proposed an OGS TV regularizer to one-dimensional signal denoising. They applied the majorization minimization (MM) method to solve their model. Their numerical experiments showed that their method can overcome staircase effects effectively. Their method has the disadvantages of the low speed of computation and the difficulty to directly extend to the two-dimensional case. However, all these methods only considered the ℓ_2_-fidelity term under Gaussian noise. To our knowledge, the methods for image deblurring and denoising under impulse noise with these regularization terms are still missing in the literature. Particularly, Liu-Huang-Liu in [27] proposed a hybrid model for image deblurring and denoising under impulse noise, but their model is not convex and it may take more time since its regularization term is the combination of TV and high-order TV with adapted parameter selection.

In this paper, inspired by the works from [40] and [41], we propose a new model for images deblurring under impulse noise by setting *ψ* in (4) to be the OGS-TV functional. In this model we first extend the OGS-TV functional in [41] to general two-dimensional case as a new regularization term, then consider an ℓ_1_-fidelity term for application in images deblurring under impulse noise. Moreover, we impose a box constraint to the proposed model to obtain more accurate solutions similarly as [26]. Using the technique of variable substitution, we provide an efficient algorithm to solve the model under the framework of ADMM. We use an inner loop which is nested inside the majorization minimization (MM) iteration for the subproblem of the proposed method. Our main contributions are firstly combining the former three parts together in one model in application and making the 2D TV with OGS case be more easily solved than the 1D case in [41]. According to a brief explanation of our model and our numerical results, we can observe that our new model using the OGS-TV regularizer coincides to maintain the edge-preserving property of TV methods and overcome the staircase effects, which is similar as total generalized variation [39]. In addition, the numerical results also show that our method is very effective and competitive with other TV-based methods, such as Chan *et al*.’s method [26] and Guo *et al*.’s method [8], especially on getting higher PSNR and better visual quality.

The outline of the rest of this paper is as follows. In the next section, we will briefly introduce the definition of the OGS regularization functional. We will also review the MM method and ADMM, which are used in our proposed method. In Section 3, we propose the OGS-TV based model for recovering images under blur and impulse noise. We also provide the efficient solving algorithm in this section. The numerical results are given in Section 4. Finally, we conclude this paper in Section 5.

## Some Preliminaries

### OGS-TV

In [41], the authors denoted a *K*-point group (*K* denotes the group size) of the vector *t* ∈ ℝ^*n*^ by

$$\begin{array}{c} t_{i,K}=\left[t\left(i\right),t\left(i+1\right),\cdots ,t\left(i+K-1\right)\right]\in {\mathbb{R}}^{K}. \end{array}$$ (5)

Note that *t*_*i*,*K*_ can be seen as a block of *K* contiguous samplings of *t* staring at index *i*. With the notation (5), a group sparsity regularizer for one-dimensional case is defined in [41] as

$$\begin{array}{c} \zeta \left(t\right)=\sum_{i=1}^{n}{∥t_{i,K}∥}_{2}. \end{array}$$ (6)

Similarly, we can define a *K*-square-point group of a two-dimensional signal such as images considered in this work *v* ∈ ℝ^*n*^2^^, where vector *v* is stacked in column-wisely, in other words, the (*i*, *j*)th entry of a matrix is assigned to be the ((*j* − 1)*n* + *i*)th entry of the vector *v*. Clearly,

$$\begin{array}{ccc} {\tilde{v}}_{i,j,K,K} & = & [\begin{array}{cccc} v_{i-K_{l},j-K_{l}} & v_{i-K_{l},j-K_{l}+1} & \cdots & v_{i-K_{l},j+K_{r}}\\ v_{i-K_{l}+1,j-K_{l}} & v_{i-K_{l}+1,j-K_{l}+1} & \cdots & v_{i-K_{l}+1,j+K_{r}}\\ \vdots & \vdots & \ddots & \vdots \\ v_{i+K_{r},j-K_{l}} & v_{i+K_{r},j-K_{l}+1} & \cdots & v_{i+K_{r},j+K_{r}} \end{array}]\in {\mathbb{R}}^{K\times K}, \end{array}$$ (7)

where $K_{l}=[\frac{K-1}{2}]$, $K_{r}=[\frac{K}{2}]$ and [*x*] denotes the largest integer less than or equal to *x*. The group size is denoted by *K* ^2^. Note that ${\tilde{v}}_{i,j,K,K}$ can be seen as a square block of *K* × *K* contiguous samplings of *v* with the center at index (*i*, *j*). Here we choose a group entries around the objective point rather than a group following the objective point like one-dimensional in [41] because of the faster and easier computation in the experiments. Moreover, to the best of our knowledge, the former is much better than the latter in image restoration because the pixels in image are related to or depended on all the ambient pixels rather than partial surrounding pixels. Let *v*_*i*,*j*,*K*,*K*_ be a *K* ^2^-vector obtained by arranging the *K* × *K* elements of ${\tilde{v}}_{i,j,K,K}$ in lexicographic order. This notation also remains valid throughout the paper unless otherwise specified. Then the overlapping group sparsity functional of the two-dimensional array can be defined by

$$\begin{array}{c} \varphi \left(v\right)=\sum_{i=1}^{n}\sum_{j=1}^{n}{∥v_{i,j,K,K}∥}_{2}. \end{array}$$ (8)

From the definition above, we can easily get that this function is convex. Consequently, we define the regularization functional *ψ* in (4) to be the form

$$\begin{array}{c} \psi \left(f\right)=\varphi \left(\nabla_{x}f\right)+\varphi \left(\nabla_{y}f\right). \end{array}$$ (9)

We call the regularizer *ψ* in (9) as the OGS anisotropic TV functional because we handle the ∇_*x*_ *f* and ∇_*y*_ *f* separately (while the isotropic TV is defined differently by ${\left\|f\right\|}_{ITV}=\sum_{i}^{n^{2}}\sqrt{{\left(\nabla_{x}f\right)}_{i}^{2}+{\left(\nabla_{y}f\right)}_{i}^{2}}$), and call the corresponding convex minimization model (4) L1-OGS-ATV.

### The MM method

The MM method is an asymptotical method in solving optimization problems, for instance, a minimization problem of the form as follows,

$$\begin{array}{c} \min_{v}P\left(v\right)=\{\frac{\alpha }{2}{∥v-v_{0}∥}_{2}^{2}+\varphi \left(v\right)\},v\in {\mathbb{R}}^{n^{2}}, \end{array}$$ (10)

where *α* is a positive parameter and the functional *φ* is defined in (8). The point of the MM method is that, instead of directly solving the difficult minimization problem *P*(*v*), the MM approach solves a sequence of easier optimization problems *Q*(*v*, *v*_*k*_) (*k* = 0, 1, 2, …) firstly and then manages to get the minimizer of *P*(*v*). Generally, an MM iterative algorithm for minimizing *P*(*v*) has the form

$$\begin{array}{c} v^{k+1}=\arg\min_{v}Q\left(v,v^{k}\right), \end{array}$$ (11)

where *Q*(*v*, *v*′) ≥ *P*(*v*) for all *v*, *v*′, and *Q*(*v*, *v*) = *P*(*v*), i.e., each functional *Q*(*v*, *v*′) is a majorizor of *P*(*v*). When *P*(*v*) is convex, then under the former conditions, the sequence *v* ^*k*^ produced by (11) converges to the minimizer of *P*(*v*) [42, 43].

We aim to solve the special problem (10), and it is obvious that *P*(*v*) in (10) is convex. Therefore, the MM approach is available for solving the problem (10). While we were concluding this manuscript, we became aware of very recent related work in [44]. The authors in [44] have studied the problem (10) elaborately, which is a subproblem of our method. Moreover, for the sake of completeness, we briefly introduce the solving method here by our way independently.

First of all, to derive an efficient algorithm with the MM scheme for solving the problem (10), we want to find a majorizor of *P*(*v*). Here, we only need to find a majorizor of *φ*(*v*) because of the simple enough quadratic term of the first term in (10). Note that

$$\begin{array}{c} \frac{1}{2}(\frac{1}{{∥u∥}_{2}}{∥v∥}_{2}^{2}+{∥u∥}_{2})\geq {∥v∥}_{2}, \end{array}$$ (12)

for all *v* and *u* ≠ 0 (*u*, *v* ∈ ℝ^*n*^2^^) with equality when *u* = *v*. Substituting each group of *φ*(*v*) into (12) and summing them, we get a majorizor of *φ*(*v*)

$$\begin{array}{c} S\left(v,u\right)=\frac{1}{2}\sum_{i=1}^{n}\sum_{j=1}^{n}[\frac{1}{∥u_{i,j,K,K}{∥}_{2}}∥v_{i,j,K,K}{∥}_{2}^{2}+{∥u_{i,j,K,K}∥}_{2}], \end{array}$$ (13)

with

$$\begin{array}{c} S(v,u)\geq \varphi (v),S(u,u)=\varphi (u), \end{array}$$ (14)

provided ‖*v*_*i*,*j*,*K*,*K*_‖ ≠ 0 for all *i*, *j*. After simple calculation, *S*(*v*, *u*) can be rewritten as

$$\begin{array}{c} S\left(v,u\right)=\frac{1}{2}{∥\Lambda \left(u\right)v∥}_{2}^{2}+C\left(u\right), \end{array}$$ (15)

where *C*(*u*) is independent of *v*, and Λ(*u*) is a diagonal matrix with each diagonal component

$${\left[\Lambda (u)\right]}_{m,m}=\sqrt{\sum_{i=-K_{l}}^{K_{r}}\sum_{j=-K_{l}}^{K_{r}}{\left[\sum_{k_{1}=-K_{l}}^{K_{r}}\sum_{k_{2}=-K_{l}}^{K_{r}}|u_{m-i+k_{1},m-j+k_{2}}{|}^{2}\right]}^{-\frac{1}{2}}},$$ (16)

with *m* = 1, 2, ⋯, *n* ^2^. The entries of Λ can be easily computed by using Matlab built-in function conv2. Then a majorizor of *P*(*v*) can be easily given by

$$\begin{array}{c} Q\left(u,v\right)=\frac{\alpha }{2}∥v-v_{0}{∥}_{2}^{2}+S\left(v,u\right)=\frac{\alpha }{2}∥v-v_{0}{∥}_{2}^{2}+\frac{1}{2}{∥\Lambda \left(u\right)v∥}_{2}^{2}+C\left(u\right), \end{array}$$ (17)

with *Q*(*v*, *u*) ≥ *P*(*v*) for all *u*, *v*, and *Q*(*u*, *u*) = *P*(*u*). To minimize *P*(*v*), the MM aims to iteratively solve

$$\begin{array}{c} v^{k+1}=\arg\min_{v}\frac{\alpha }{2}∥v-v_{0}{∥}_{2}^{2}+\frac{1}{2}{∥\Lambda \left(v^{k}\right)v∥}_{2}^{2},k=1,2,\cdots , \end{array}$$ (18)

with the solution

$$\begin{array}{c} {\hat{v}}^{k+1}={\left(I+\frac{1}{\alpha }\Lambda^{2}\left(v^{k}\right)\right)}^{-1}v_{0},k=1,2,\cdots . \end{array}$$ (19)

where *I* is an identity matrix with the same size of Λ(*v* ^*k*^). We can easily get that Λ^2^(*v* ^*k*^) is also a diagonal matrix with each diagonal component [Λ^2^(*v* ^*k*^)]_*m*,*m*_ equaling to the form of removing the out root of the right term of (16). Moreover, the inversion of the matrix $I+\frac{1}{\alpha }\Lambda^{2}(v^{k})$ can be computed very efficiently since it only requires simple componentwise calculation. Therefore, we obtain the Algorithm 1 for solving the problem (10).

**Algorithm 1** The MM method for solving (10)

1. ***initialization***: Starting point *v* = *v*_0_, *α*, group size *K* ^2^, $K_{l}=[\frac{K-1}{2}]$,

$K_{r}=[\frac{K}{2}]$, *ϵ*, Maximum inner iterations *NIt*, *k* = 0.

2. ***iteration***:

 Do

$\begin{array}{cc} \;\;{\left[\Lambda^{2}\left(v^{k}\right)\right]}_{m,m} & \;=\sum_{i=-K_{l}}^{K_{r}}\sum_{j=-K_{l}}^{K_{r}}{\left[\sum_{k_{1}=-K_{l}}^{K_{r}}\sum_{k_{2}=-K_{l}}^{K_{r}}{|v_{m-i+k_{1},m-j+k_{2}}^{k}|}^{2}\right]}^{-\frac{1}{2}},\\ v^{k+1} & \;=\;{\left(I+\frac{1}{\alpha }\Lambda^{2}\left(v^{k}\right)\right)}^{-1}v_{0},\\ k & \;=\;k+1, \end{array}$

 until ‖*v* ^*k*+1^ − *v* ^*k*^‖_2_/‖*v* ^*k*^‖_2_ < *ϵ* or *k* > *NIt*.

3. ***get** v^k^*.

### Variable splitting and ADMM

Consider an unconstrained optimization problem in which the objective function is the sum of two functions as

$$\begin{array}{c} \min\phi_{1}\left(x_{1}\right)+\phi_{2}\left(x_{2}\right),s.\;t.\;A_{1}x_{1}+A_{2}x_{2}=b,x_{i}\in \chi_{i},i=1,2, \end{array}$$ (20)

where *ϕ_i_*: ℝ^*n_i_*^ → ℝ are closed proper convex functions, *χ_i_* ⊆ ℝ^*n_i_*^ are closed convex sets, *A_i_* ∈ ℝ^*l*×*n_i_*^, and *b* ∈ ℝ^*l*^ is a given vector. The augmented Lagrangian function ([28]) of (20)

$$\begin{array}{ccc} 𝓛(x_{1},x_{2},\lambda ) & = & \phi_{1}\left(x_{1}\right)+\phi_{2}\left(x_{2}\right)-\lambda^{T}\left(A_{1}x_{1}+A_{2}x_{2}-b\right)+\frac{\beta }{2}{∥A_{1}x_{1}+A_{2}x_{2}-b∥}_{2}^{2}\\ & = & \phi_{1}\left(x_{1}\right)+\phi_{2}\left(x_{2}\right)+\frac{\beta }{2}{∥A_{1}x_{1}+A_{2}x_{2}-b-\frac{\lambda }{\beta }∥}_{2}^{2}+C \end{array}$$ (21)

where *λ* ∈ ℝ^*l*^ is the Lagrange multiplier, *β* is a penalty parameter which controls the linear constraint, and *C* does not depend on *x*_1_, *x*_2_. The idea of ADMM is to find a saddle point of 𝓛. Usually, ADMM consists in alternated minimizing 𝓛 on *x*_1_, *x*_2_, *λ*, for instance, minimizing 𝓛 with respect to *x*_1_ by fixing *x*_2_ and *λ*. That delivers to the following simple but powerful algorithm classic ADMM.

**Algorithm 2** Classic ADMM for the minimization problem (20)

***initialization***: Starting point$x_{1}^{0}$, $x_{2}^{0}$, *λ* ^0^, *β*.

***iteration***:

$\begin{array}{ccc} x_{1}^{k+1} & = & \arg\;\min\;\phi_{1}(x_{1})+\frac{\beta }{2}\|A_{1}x_{1}+A_{2}x_{2}^{k}-b-\frac{\lambda^{k}}{\beta }{\|}_{2}^{2},\\ x_{2}^{k+1} & = & \arg\;\min\;\phi_{2}(x_{2})+\frac{\beta }{2}\|A_{1}x_{1}^{k+1}+A_{2}x_{2}-b-\frac{\lambda^{k}}{\beta }{\|}_{2}^{2},\\ \lambda^{k+1} & = & \lambda^{k}-\beta (A_{1}x_{1}^{k+1}+A_{2}x_{2}^{k+1}-b),\;\;\;\;\;\;\;\;\;\;\\ k & = & k+1, \end{array}$

***until a stopping criterion is satisfied.***

According to [12], we can see classic ADMM is convergent because of the nonexpansive and absolute summable properties of the *x*_1_ and *x*_2_ subproblems. More details can be found in [12, 28–31]. However, the convergence speed is not too fast. In order to speed the convergence, we can introduce a step length parameter *γ* for updating the multiplier [29, 32, 33]. The algorithm framework is outlined as follows called general ADMM.

**Algorithm 3** General ADMM for the minimization problem (20)

***initialization***: Starting point$x_{1}^{0}$, $x_{2}^{0}$, *λ* ^0^, *β*.

***iteration***:

$\begin{array}{ccc} x_{1}^{k+1} & = & \arg\;\min\;\phi_{1}(x_{1})+\frac{\beta }{2}\|A_{1}x_{1}+A_{2}x_{2}^{k}-b-\frac{\lambda^{k}}{\beta }{\|}_{2}^{2},\\ x_{2}^{k+1} & = & \arg\;\min\;\phi_{2}(x_{2})+\frac{\beta }{2}\|A_{1}x_{1}^{k+1}+A_{2}x_{2}-b-\frac{\lambda^{k}}{\beta }{\|}_{2}^{2},\\ \lambda^{k+1} & = & \lambda^{k}-\gamma \beta (A_{1}x_{1}^{k+1}+A_{2}x_{2}^{k+1}-b),\;\;\;\;\;\;\;\;\;\;\\ k & = & k+1, \end{array}$

***until a stopping criterion is satisfied.***

Here, *γ* > 0 is also called a relax parameter. In fact, if *γ* = 1, general ADMM is classic ADMM. From [29, 32, 33], general ADMM is convergent if $\gamma \in (0,(\sqrt{5}+1)/2)$. Moreover, *γ* = 1.618 makes it converge noticeably faster than *γ* = 1. Therefore, we set *γ* = 1.618 in our work.

## Proposed Method

With the definition of (9), we will consider a minimization problem of the form (L1-OGS-ATV)

$$\begin{array}{c} \min_{f}\varphi \left(\nabla_{x}f\right)+\varphi \left(\nabla_{y}f\right)+\mu {∥Hf-g∥}_{1}. \end{array}$$ (22)

Note that for any true digital image, its pixel values can attain only a finite number of values. Hence, it is natural to require all pixel values of the restored image to lie in a certain interval [*a*, *b*], see [26] for more details. For example, for 8-bit images, we would like to restore them in a dynamic range [0, 255]. More in general, with the easy computation and the certified results in [26], we only consider all the images located on the range [0, 1]. Therefore, the images we mentioned all lie in the interval [0, 1]. We define a projection operator 𝓟_Ω_ on the set Ω = {*f* ∈ ℝ^*n*×*n*^∣0 ⩽ *f* ⩽ 1},

$$\begin{array}{c} {𝓟}_{\Omega }{\left(f\right)}_{i,j}=\{\begin{array}{cc} 0, & f_{i,j}<0,\\ f_{i,j}, & f_{i,j}\in \left[0,1\right],\\ 1, & f_{i,j}>1. \end{array} \end{array}$$ (23)

Similarly as [26], we will solve the problem

$$\begin{array}{c} \min_{f\in \Omega }\varphi \left(\nabla_{x}f\right)+\varphi \left(\nabla_{y}f\right)+\mu {∥Hf-g∥}_{1}. \end{array}$$ (24)

We refer to this model as CL1-OGS-ATV. Obviously, this model is also convex.

Particularly, each term of the model (24) has the properties of additivity and separability. Therefore, we can rewrite it as follows (under the periodic boundary conditions),

$$\begin{array}{cc} & \min_{f\in \Omega }\varphi \left(\nabla_{x}f\right)+\varphi \left(\nabla_{y}f\right)+\mu {∥Hf-g∥}_{1}\\ = & \min_{f\in \Omega }\sum_{i=1}^{n}\sum_{j=1}^{n}{∥{\left(\nabla_{x}f\right)}_{i,j,K,K}∥}_{2}+\sum_{i=1}^{n}\sum_{j=1}^{n}{∥{\left(\nabla_{y}f\right)}_{i,j,K,K}∥}_{2}\\ & +\;\mu \sum_{i=1}^{n}\sum_{j=1}^{n}\frac{1}{K\times K}{∥{\left(Hf-g\right)}_{i,j,K,K}∥}_{1}\\ = & \min_{f\in \Omega }\sum_{i=1}^{n}\sum_{j=1}^{n}({∥{\left(\nabla_{x}f\right)}_{i,j,K,K}∥}_{2}+{∥{\left(\nabla_{y}f\right)}_{i,j,K,K}∥}_{2}+\frac{\mu }{K\times K}{∥{\left(Hf-g\right)}_{i,j,K,K}∥}_{1}). \end{array}$$ (25)

From the Eq (25), we can observe that our model (24) can be seen as a combination of *n* × *n* coupled subproblems, which are all (*i*, *j*) terms of the last line in (25). Each subproblem is approximate to the original TV regularization model (4) with *ψ*(*f*) = ‖*f*‖_*TV*_ in a local region, which has the edge-preserving property. More details please refer to [45].

Therefore, our model (24) can coincide to 1) maintain the edge-preserving property of TV methods, and 2) have the property of smoothing the local regions, which can be seen as overcoming the staircase effects. However, we did not solve our model decoupled as (25) in this work, so we choose the following algorithm to solve (24) based on ADMM.

For the model (24), by introducing new auxiliary variables *v*_*x*_, *v*_*y*_, *z*, *w*, we transform the minimization problem (24) to the equivalent constrained minimization problem

$$\begin{array}{c} \min_{w\in \Omega ,f,z,v_{x},v_{y}}\{\varphi \left(v_{x}\right)+\varphi \left(v_{y}\right)+\mu {∥z∥}_{1}:\;z=Hf-g,v_{x}=\nabla_{x}f,v_{y}=\nabla_{y}f,w=f\}. \end{array}$$ (26)

Note that the constraint is now imposed on *w* instead of *f*. The augmented Lagrangian function of (26) is

$$\begin{array}{ccc} & & 𝓛(v_{x},v_{y},z,w,f;\lambda_{1},\lambda_{2},\lambda_{3},\lambda_{4})=\\ & & \varphi \left(v_{x}\right)-\lambda_{1}^{T}(v_{x}-\nabla_{x}f)+\frac{\beta_{1}}{2}{∥v_{x}-\nabla_{x}f∥}_{2}^{2}\\ & & +\varphi \left(v_{y}\right)-\lambda_{2}^{T}(v_{y}-\nabla_{y}f)+\frac{\beta_{1}}{2}{∥v_{y}-\nabla_{y}f∥}_{2}^{2}\\ & & +{\mu ∥z∥}_{1}-\lambda_{3}^{T}(z-(Hf-g))+\frac{\beta_{2}}{2}{∥z-\left(Hf-g\right)∥}_{2}^{2}\\ & & -\lambda_{4}^{T}\left(w-f\right)+\frac{\beta_{3}}{2}{∥w-f∥}_{2}^{2}, \end{array}$$ (27)

where *β*_1_, *β*_2_, *β*_3_ > 0 are penalty parameters and *λ*_1_, *λ*_2_, *λ*_3_, *λ*_4_ ∈ ℝ^*n*^2^^ are the Lagrange multipliers. According to the scheme of general ADMM mentioned above (Algorithm 3), for a given ($v_{x}^{k},v_{y}^{k},z^{k},w^{k},f^{k}$; $\lambda_{1}^{k},\lambda_{2}^{k},\lambda_{3}^{k},\lambda_{4}^{k}$), the next iteration $(v_{x}^{k+1},v_{y}^{k+1},z^{k+1},w^{k+1},f^{k+1}$; $\lambda_{1}^{k+1},\lambda_{2}^{k+1},\lambda_{3}^{k+1},\lambda_{4}^{k+1})$ is generated as follows:

1. Fix *f* = *f* ^*k*^, $\lambda_{1}=\lambda_{1}^{k},\lambda_{2}=\lambda_{2}^{k},\lambda_{3}=\lambda_{3}^{k},\lambda_{4}=\lambda_{4}^{k}$, *z* = *z* ^*k*^, *w* = *w* ^*k*^, and minimize (27) with respect to *v*_*x*_ and *v*_*y*_. The minimizers are obtained by
  $$\begin{array}{ccc} v_{x}^{k+1} & = & \arg\min\varphi \left(v_{x}\right)-{\lambda_{1}^{k}}^{T}\left(v_{x}-\nabla_{x}f^{k}\right)+\frac{\beta_{1}}{2}{∥v_{x}-\nabla_{x}f^{k}∥}_{2}^{2}\\ & = & \arg\min\varphi \left(v_{x}\right)+\frac{\beta_{1}}{2}{∥v_{x}-\nabla_{x}f^{k}-\frac{\lambda_{1}^{k}}{\beta_{1}}∥}_{2}^{2}, \end{array}$$ (28)
  $$\begin{array}{ccc} v_{y}^{k+1} & = & \arg\min\varphi \left(v_{y}\right)-{\lambda_{2}^{k}}^{T}\left(v_{y}-\nabla_{y}f^{k}\right)+\frac{\beta_{1}}{2}{∥v_{y}-\nabla_{y}f^{k}∥}_{2}^{2}\\ & = & \arg\min\varphi \left(v_{y}\right)+\frac{\beta_{1}}{2}{∥v_{y}-\nabla_{y}f^{k}-\frac{\lambda_{2}^{k}}{\beta_{1}}∥}_{2}^{2}. \end{array}$$ (29)
  It is obvious that problems (28) and (29) match the framework of the problem (10), thus the solutions of (28) and (29) can be obtained by using Algorithm 1, respectively.
2. Compute *z* ^*k*+1^.
  $$\begin{array}{ccc} z^{k+1} & = & {\arg\min\mu ∥z∥}_{1}-{\lambda_{3}^{k}}^{T}(z-(Hf^{k}-g))+\frac{\beta_{2}}{2}{∥z-\left(Hf^{k}-g\right)∥}_{2}^{2}\\ & = & {\arg\min\mu ∥z∥}_{1}+\frac{\beta_{2}}{2}{∥z-\left(Hf^{k}-g\right)-\frac{\lambda_{3}^{k}}{\beta_{2}}∥}_{2}^{2}. \end{array}$$
  The minimization with respect to *z* can be given by the well-known Shrinkage [20] explicitly:
  $$\begin{array}{c} z^{k+1}=\mathrm{sgn}\{Hf^{k}-g+\frac{\lambda_{3}^{k}}{\beta_{2}}\}\circ \max\{|Hf^{k}-g+\frac{\lambda_{3}^{k}}{\beta_{2}}|-\frac{\mu }{\beta_{2}},0\}, \end{array}$$ (30)
  where ∣ ⋅ ∣, sgn and “∘” represent the componentwise absolute value, signum function, and componentwise product, respectively.
3. Compute *w* ^*k*+1^.
  $$\begin{array}{ccc} w^{k+1} & = & \arg\min-{\lambda_{4}^{k}}^{T}\left(w-f^{k}\right)+\frac{\beta_{3}}{2}{∥w-f^{k}∥}_{2}^{2}\\ & = & \arg\min\frac{\beta_{3}}{2}{∥w-f^{k}-\frac{\lambda_{4}^{k}}{\beta_{3}}∥}_{2}^{2}. \end{array}$$
  The minimizer is given explicitly by
  $$\begin{array}{c} w^{k+1}={𝓟}_{\Omega }[f^{k}+\frac{\lambda_{4}^{k}}{\beta_{3}}]. \end{array}$$ (31)
4. Compute *f* ^*k*+1^ by solving the normal equation
  $$\begin{array}{ccc} & & \left(\beta_{1}\left(\nabla_{x}^{*}\nabla_{x}+\nabla_{y}^{*}\nabla_{y}\right)+\beta_{2}H^{*}H+\beta_{3}I\right)f^{k+1}\\ & & =\nabla_{x}^{*}\left(\beta_{1}v_{x}^{k+1}-\lambda_{1}^{k}\right)+{\nabla_{y}}^{*}\left(\beta_{1}v_{y}^{k+1}-\lambda_{2}^{k}\right)+H^{*}\left(\beta_{2}z^{k+1}-\lambda_{3}^{k}\right)\\ & & +\beta_{2}H^{*}g+\beta_{3}\left(w^{k+1}-\frac{\lambda_{4}^{k}}{\beta_{3}}\right), \end{array}$$ (32)
  where “*” denotes the conjugate transpose, see [13] for more details. Since all the parameters are positive, the coefficient matrix in (32) are always invertible and symmetric positive definite. In addition, note that *H*, ∇_*x*_, ∇_*y*_ and their conjugate transpose have BCCB structure under periodic boundary conditions. We know that the computations with BCCB matrix can be very efficient by using fast Fourier transforms (FFTs).
5. Update the multipliers via
  $$\begin{array}{c} \left\{ \begin{array}{ccc} \lambda_{1}^{k+1} & = & \lambda_{1}^{k}-\gamma \beta_{1}\left(v_{x}^{k+1}-\nabla_{x}f^{k+1}\right),\\ \lambda_{2}^{k+1} & = & \lambda_{2}^{k}-\gamma \beta_{1}\left(v_{y}^{k+1}-\nabla_{y}f^{k+1}\right),\\ \lambda_{3}^{k+1} & = & \lambda_{3}^{k}-\gamma \beta_{2}\left(z^{k+1}-\left(Hf^{k+1}-g\right)\right),\\ \lambda_{4}^{k+1} & = & \lambda_{4}^{k}-\gamma \beta_{3}\left(w^{k+1}-f^{k+1}\right). \end{array}\right. \end{array}$$ (33)

Based on the discussions above, we present the algorithm on ADMM using inner MM iteration for solving the convex CL1-OGS-ATV model (24) shown as Algorithm 4.

**Algorithm 4** CL1-OGS-ATV-ADM4 for the minimization problem (24)

***initialization***:

 Starting point $v_{x}^{0}=v_{y}^{0}=g$, *k* = 0, *β*_1_, *β*_2_, *β*_3_, *γ*, *μ*, group size *K* × *K*,

$\lambda_{i}^{0}=0$, *i* = 1, 2, 3, 4, Maximum inner iterations *NIt*.

***iteration***:

 1. Compute $v_{x}^{k+1}$ and $v_{y}^{k+1}$ according to (28) and (29).

 2. Compute *z* ^*k*+1^ according to (30).

 3. Compute *w* ^*k*+1^ according to (31).

 4. Compute *f* ^*k*+1^ by solving (32).

 5. update $\lambda_{i}^{0}=0,i=1,2,3,4$ according to (33).

 6. *k* = *k* + 1.

***until a stopping criterion is satisfied.***

CL1-OGS-ATV-ADM4 is a special form of general ADMM for the case with two blocks of variables (*v*_*y*_, *v*_*x*_, *w*, *z*) and *f*. If the Step (1) of Algorithm 4 can be solved exactly, the convergence for CL1-OGS-ATV-ADM4 can be guaranteed. In this case, if the relax parameter $\gamma \in (0,\frac{\sqrt{5}+1}{2})$, Algorithm 4 is convergent, more details can be referred to [29, 30, 32, 33]. Besides, although step (1) of Algorithm 4 can not be solved exactly, we can find a convergent series to ensure the convergence as [12]. Particularly, our numerical experiments verify the convergence of Algorithm 4.

## Numerical Results

In this section, we present several numerical results to illustrate the performance of the proposed method. We compare our method CL1-OGS-ATV-ADM4 (“Ours” for short) with other state-of-the-art methods, Chan *et al*.’s ADM2CTVL1 proposed in [26] (“CTY” for short, Algorithm 2 in [26] for the constrained TV-L1 model), Guo *et al*.’s fast ℓ_1_-TV proposed in [8] (“GLN” for short) and a high-order method derived by ourselves.

All experiments are carried out on Windows 7 32-bit and Matlab 2010a running on a desktop equipped with an Intel Core i3-2130 CPU with 3.4 GHz and 3.4 GB of RAM.

The quality of the restoration results is measured quantitatively by using the peak signal-to-noise ratio (PSNR) in decibel (dB) and the relative error (ReE):

$$\begin{array}{c} \mathrm{PSNR}=10\log_{10}\frac{n^{2}{\mathrm{Max}}_{I}^{2}}{∥f-\bar{f}{∥}_{2}^{2}},\;\mathrm{ReE}=\frac{∥f-\bar{f}{∥}_{2}}{∥\bar{f}{∥}_{2}}, \end{array}$$

where $\bar{f}$ and *f* denote the original and restored images respectively, and Max_*I*_ represents the maximum possible pixel value of the image. In our experiments, Max_*I*_ = 1. The stopping criterion used in our work is set to be

$$\begin{array}{c} \frac{|{𝓕}^{k+1}-{𝓕}^{k}|}{|{𝓕}^{k}|}<10^{-5}, \end{array}$$ (34)

where 𝓕^*k*^ is the objective function value of the respective model in the *k*th iteration, which is

$$\begin{array}{c} {𝓕}^{k}=\varphi \left(\nabla_{x}f^{k}\right)+\varphi \left(\nabla_{y}f^{k}\right)+\mu {∥Hf^{k}-g∥}_{1}. \end{array}$$ (35)

The stopping criterions of CTY (same as ours) and GLN are set to default as their literature mentioned.

**Fig 1:**
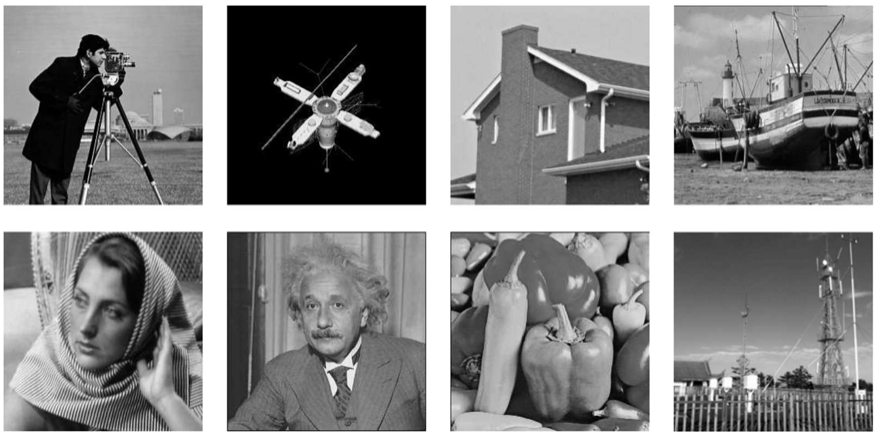
Original images. Top row: from left to right, (a) Cameraman, (b) Satellite, (c) House, (d) Boat. Bottom row: from left to right, (e) Barbara, (f) Einstein, (g) Peppers, (h) Weatherstation.

All the test images are shown in Fig 1, seven 256-by-256 images as: (a) Cameraman.tif, (b) Satellite.pgm, (c) House.png, (d) Boat.pgm, (e) Barbara.tiff, (f) Einstein.pgm, (g) Peppers.png and one 460-by-460 image (h) Weatherstation.tif For the sake of simplicity, the pixel values in all of our tests are lied in [0, 1] which have been explained above.

**Remark 1**. The image “House” is downloaded from http://sipi.usc.edu/database/database.php?volume=misc&image=5top. The image “Satellite” is from “restore tools” downloaded from http://www.mathcs.emory.edu/~nagy/RestoreTools/. The images “Cameraman”, “Boat”, “Barbara”, “Einstein” and “Peppers” are from http://decsai.ugr.es/cvg/dbimagenes/. The image “Weatherstation” is photographed by one of the authors–Jun Liu in 2011, which can be found from https://www.flickr.com/photos/98566316@N06/14092821600/in/photostream/. The versions of the images in our paper are other special formats which are converted by Photoshop from the sources above.

We set the penalty parameters *β*_1_ = 1, *β*_2_ = 500, *β*_3_ = 1, and relax parameter *γ* = 1.618 throughout all the experiments. Three blur kernels are generated by Matlab built-in function (i) fspecial(‘gaussian’, 7, 5) for 7 × 7 Gaussian blur with standard deviation 5, (ii) fspecial(‘gaussian’, 15, 5) for 15 × 15 Gaussian blur with standard deviation 5 and (iii) fspecial(‘average’, 7) for 7 × 7 average blur. We generate all blurring effects using the Matlab built-in function imfilter(I, psf, ‘circular’, ‘conv’) under periodic boundary conditions with “I” the original image and “psf” the blur kernel. We generate all noise effects by Matlab built-in function imnoise(B, ‘salt & pepper’, level) with “B” the blurred image and fix the same random matrix for different methods. We only consider the salt-and-pepper noise in our experiments, since the variation method is easy to extend to the random value noise case.

### Study on the rest parameters

**Fig 2:**
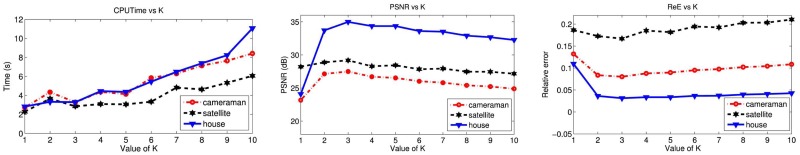
Results of our proposed method depending on group size parameter *K*. The test images (“Cameraman”, “Satellite”, “House”) are blurred by Gaussian blur kernel with 7 × 7 and standard deviation 5 and corrupted by 40% salt-and-pepper noise. From left to right, results on CPU time, PSNR, ReE respectively.

Firstly, we set the group size parameter *K* = 3 to find a good maximum inner iterations *NIt*. Our experiments are on the image “Cameraman” blurred by Gaussian blur kernel with 7 × 7 and standard deviation 5 and corrupted by 40% salt-and-pepper noise. The results are shown in Table 1. From Table 1, we can choose maximum inner iterations *NIt* = 5 for the best. Here we find that the larger the inner iteration *NIt* (> 5) is, the lower PSNR values are, which is a drawback for the selection of *NIt*. This is because we do not tune the other parameters to be best for different *NIt*. After we test more similar tests, and due to the CPU time and little error between different selections of *NIt*, we choose *NIt* = 5 for balance with the following experiments. Then we fix *NIt* = 5 and repeat more experiments for choosing a good group size parameter *K*. We operate the three 256-by-256 images (a) “Cameraman”, (b) “Satellite”, and (c) “House” for this best option of parameter *K*. The results are shown in Fig 2. From the figure, we can see that *K* = 3 is better for all the tests both on CPU time and PSNR. Therefore, we fix that *NIt* = 5 and *K* = 3 for balance in our work.

**Table 1:** PSNR (dB) and time (s) depending on maximum inner iterations *NIt* on the image “Cameraman” with Gaussian blur kernel 7 × 7 and standard deviation 5 and 40% salt-and-pepper noise.

**NIt**	1	3	5	7	10	20	50	100	200	1000
**PNSR**	21.38	27.44	27.50	27.36	27.22	27.00	26.87	26.85	26.84	26.83
**Time**	3.245	2.868	3.267	4.274	6.022	10.87	23.84	45.54	89.30	1965

**Fig 3:**
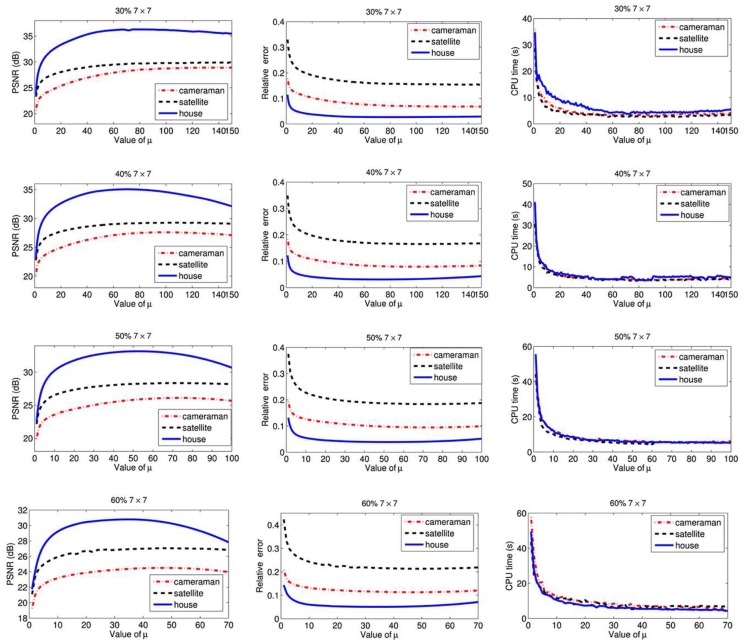
Results of our proposed method depending on regularization parameter *μ*. The tests are on the images “Cameraman”, “Satellite”, “House” that are blurred by 7 × 7 Gaussian blur kernel with standard deviation 5 and corrupted by salt-and-pepper noise from 30% to 60%. From left to right, results on PSNR, ReE, CPU time respectively. From top to bottom, results on noise level 30%, 40%, 50%, 60% respectively.

Then, we test how to select a good regularization parameter *μ* for different images. We will point out several important advantages of our method in the following experiments. For the sake of simplicity, we focus on the above three test 256-by-256 images (a) (b) and (c). Under the Gaussian blur with 7 × 7 window size and standard deviation 5, the images corrupted by added salt-and-pepper noise from 30% to 60% are tested. In Fig 3, we plot PSNR, ReE, and Time for our algorithm against different values of the regularization parameter *μ*. Each row in Fig 3 corresponds to the four salt-and-pepper noise levels. In fact, for all *μ*, our method always gives high PSNR values. Moreover, the PSNR curves of our method are very flat, which shows that our method is stable for a wide range of *μ*, which is wider than that in [26]. That is to say, our method is more robust than the method in [26]. This is the first advantage of our method.

In addition, as far as we know, a good image restoration algorithm should satisfy the following two properties. a) It is fast and can reach good results in term of both numerical values and high visual quality. b) It is not sensitive to parameters. Our method meets the requirement of these two properties, as it obtains good restoration results with the same parameters for different images under the same blur and noise. This is the second advantage of our method. Here and in the following experiments, under the same blur and noise, we choose the same parameters for all the test images. Particularly, for the images under the Gaussian blur with 7 × 7 window size and standard deviation 5 and corrupted by salt-and-pepper noise from 30% to 60%, we set *μ* = 100, 80, 60, 40 respectively. After similar tests as Fig 3, we list all the selection rule of *μ*: for the images under the Gaussian blur with 15-by-15 window size and standard deviation 5 and corrupted by salt-and-pepper noise from 30% to 60%, *μ* = 120, 110, 100, 90 respectively; for the images under the average blur with 7 × 7 window size and corrupted by salt-and-pepper noise from 30% to 60%, *μ* = 100, 80, 60, 40 respectively.

### Comparison with CTY and GLN for the test image “Cameraman”

In this subsection, we mainly compare our proposed method to CTY and GLN for deblurring problems under salt-and-pepper noise. We use image “Cameraman” for the experiments in this subsection. Our purposes are (1) to show the improvement of PSNR and to demonstrate the efficiency of our proposed method mainly via a comparison with CTY and GLN, and (2) to illustrate that our proposed method can overcome the staircase effects effectively and get better visual quality than CTY and GLN, which is the third advantage of our method.

Firstly, we generate the blurred images by two Gaussian blurs (i) and (ii) with periodic boundary conditions as mentioned above, and then corrupt the blurred images by salt-and-pepper noise from 30% to 60%. For CTY and GLN, we have tuned the parameters manually to give the best PSNR improvement. The numerical results by the three methods are shown in Table 2. From the table, we see that both our proposed method and CTY are much faster and can get higher PSNR than GLN. Our proposed method needs the fewest iterations than the other two methods, and the time is always close to CTY. Particularly, the iterations of the GLN method always reach the maximum number of iterations which we set to be 200.

**Table 2:** Numerical comparison of the fast ℓ_1_-TV method (GLN) [8], the ADM2CTVL1 method (CTY) [26], and our proposed method (Ours) under two Gaussian blurs (Bls) (i) fspecial(‘gaussian’, 7, 5) and (ii) fspecial(‘gaussian’, 15, 5) and corrupted by salt-and-pepper noise from 30% to 60%.

**Bls**	**Noise**	**GLN**				**CTY**				**Ours**			
	**level**	**Itrs**	**PSNR**	**Time**	**ReE**	**Itrs**	**PSNR**	**Time**	**ReE**	**Itrs**	**PSNR**	**Time**	**ReE**
**(i)**	30%	200	27.34	8.30	0.0893	122	27.66	4.91	0.0787	38	28.73	3.24	0.0696
	40%	200	25.93	8.11	0.0961	102	26.63	3.91	0.0887	43	27.50	3.59	0.0802
	50%	200	24.73	8.13	0.1103	77	25.42	2.90	0.1019	49	26.00	3.92	0.0953
	60%	200	23.43	8.38	0.1281	56	24.20	2.51	0.1178	62	24.50	4.96	0.1137
**(ii)**	30%	200	24.37	8.16	0.1155	113	24.10	3.68	0.1183	37	24.52	2.92	0.1132
	40%	200	23.76	8.05	0.1235	95	23.86	3.57	0.1221	35	24.22	2.88	0.1169
	50%	200	23.46	8.11	0.1277	61	23.55	2.56	0.1246	35	23.93	2.91	0.1210
	60%	200	22.05	8.51	0.1502	55	23.12	2.17	0.1327	36	23.48	2.73	0.1274

**Fig 4:**
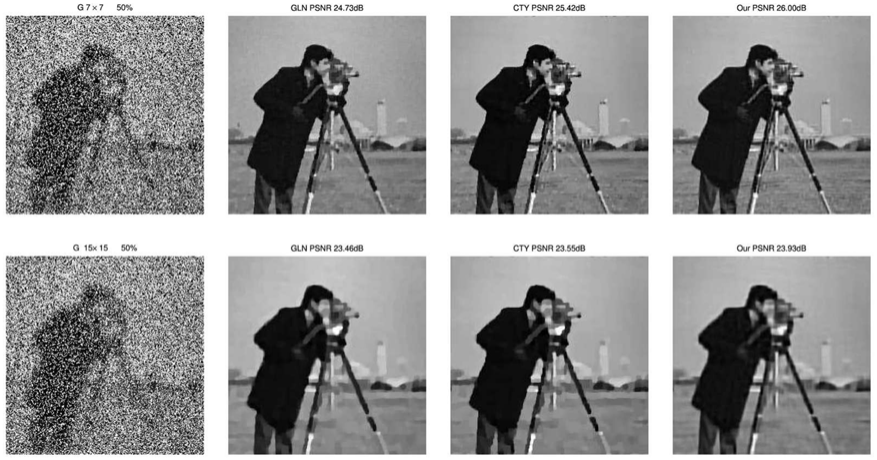
Degraded and restored images of CTY, GLN and our method. Left column: blurred and noisy images under 7 × 7 and 15 × 15 Gaussian blur with standard deviation 5 and corrupted by 50% salt-and-pepper noise. Right columns: restored images by GLN, CTY, and our proposed method respectively. The PSNR results can be found clearly from Table 2.

**Fig 5:**
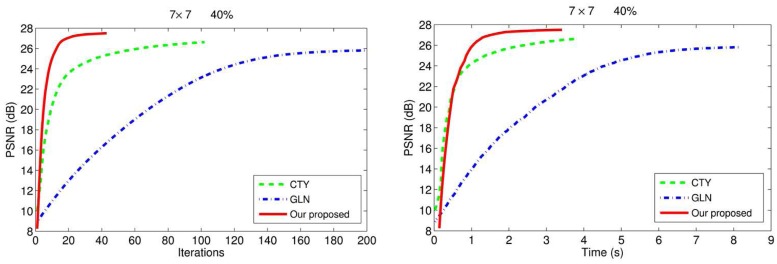
PSNR over time and iterations results of GLN, CTY, and our proposed method. Restoration of the “Cameraman” image under 7 × 7 Gaussion blur with standard deviation 5 and corrupted by 40% salt-and-pepper noise: evolution of the PSNR over time and iterations of GLN, CTY, and our proposed method.

We also show the images restored by the three methods. We display the degraded images and the restored images by three methods under two Gaussian blurs (i) and (ii) and 50% level of noise. The results are show in Fig 4. We can easily see the third advantage of our proposed method that our method can overcome the staircase effects effectively and get better visual quality than others. Moreover, we also plot the evolution of the PSNR over time and iterations for the three different methods in Fig 5 for the image blurred by 7 × 7 Gaussian blur and corrupted by 40% level salt-and-pepper noise.

From the expriments and the description in [8], the GLN method has three sensitive parameters that depend on blur, noise level and test images rather than only one sensitive regular parameter *μ* in CTY and our proposed method. Besides, the results of GLN is nearly same as FTVd, while CTY is much better than FTVd in [26]. From the above tests, we also observe that both our proposed method and CTY can get better results than GLN. Moerever, the staircase effects by GLN method is also existent. Therefore, we omit the following comparison with GLN and only list the comparison with CTY.

**Remark 2**. Here and in the following tests for CTY, we tune the regularization parameter *μ* to be optimal by checking the highest PSNR and the lowest ReE under a “for loop” of Matlab code from 1 to 70 by step length 1 for different images under different blurs and noise levels. Besides, in the experimentsunder the 15 × 15 Gaussion blur with standard deviation 5, we find that if we increase the inner penalty parameter *β*_2_ we will get higher PSNR. We do not change the parameter in our proposed method because of the good properties of a good restoration algorithm introduced above. However, in Table 2 for the CTY method under 15 × 15 Gaussion blur, we set the inner penalty parameter *β*_2_ = 50 instead of *β*_2_ = 20 in [26] for higher PSNR. For other tests following we also set this inner penalty parameter of CTY to be as default (*β*_2_ = 20) in [26].

### Comparison with CTY for other test images

In this subsection, we focus on comparisons between our proposed method and CTY for deblurring problems under salt-and-pepper noise. Fifty-six degraded test images are generated in the way similar to that in the last subsection. That is, we first generated the blurred images operating on images (b)-(h) with the periodic boundary condition by two blurs Gaussian blur (i) (also as G) and average blur (iii) (also as A), then corrupted the blurred images by salt-and-pepper noise from 30% to 60%. The parameters of our proposed method are set as above description and the parameters of CTY as Remark 2.

Conclusions similar to those in the last subsection can be made based on the results in Table 3 and Table 4. For example, our proposed method is always more accurate, with a possible improvement of more than 2.90 dB in PSNR (see image (c) with Gaussian blur and a 30% level of noise). For all images, the lower the noise level is, the better the improvement of PSNR will be. Even in high noise level, our method is more accurate than CTY by sacrificing partial time. For a further step, the iterations of our method are fewer than CTY for almost all test.

**Table 3:** Numerical comparison of CTY and our proposed method under 7 × 7 Gaussian blur with standard deviation 5 and corrupted by salt-and-pepper noise from 30% to 60%.

**Images**	**Noise**	**CTY**					**Ours**				
	**level**	***μ***	**Itrs**	**PSNR**	**Time**	**ReE**	***μ***	**Itrs**	**PSNR**	**Time**	**ReE**
**(b) Satellite**	30%	25	75	29.26	3.01	0.1653	100	31	29.78	2.65	0.1558
	40%	24	64	28.59	2.36	0.1787	80	36	29.18	2.76	0.1669
	50%	24	54	27.70	1.98	0.1978	60	40	28.28	3.32	0.1850
	60%	11	45	26.80	1.93	0.2196	40	66	27.04	4.52	0.2136
**(c) House**	30%	16	90	33.26	3.45	0.0381	100	37	36.20	2.96	0.0272
	40%	12	75	32.53	3.23	0.0414	80	42	34.99	3.45	0.0312
	50%	13	64	31.56	2.69	0.0463	60	49	33.11	3.87	0.0288
	60%	9	61	30.16	2.58	0.0545	40	62	30.79	4.27	0.0506
**(d) Boat**	30%	25	129	28.30	5.65	0.0720	100	35	29.95	2.70	0.0595
	40%	24	111	27.24	4.27	0.0813	80	36	28.77	2.71	0.0682
	50%	18	82	26.13	3.21	0.0924	60	42	27.23	3.20	0.0814
	60%	10	61	24.87	2.54	0.1069	40	58	25.57	4.46	0.0985
**(e) Barbara**	30%	35	125	25.57	5.21	0.0985	100	33	27.00	3.24	0.0835
	40%	30	87	24.77	4.77	0.1080	80	41	25.92	3.95	0.0946
	50%	21	84	24.14	3.70	0.1162	60	34	24.87	3.24	0.1067
	60%	6	58	23.62	2.37	0.1234	40	61	23.94	5.01	0.1189
**(f) Einstein**	30%	18	106	31.67	4.60	0.0583	100	36	32.87	3.60	0.0509
	40%	16	80	30.87	3.42	0.0640	80	36	32.04	3.28	0.0559
	50%	16	75	29.88	3.21	0.0718	60	47	30.80	4.04	0.0645
	60%	12	64	28.13	2.54	0.0878	40	62	28.59	5.27	0.0832
**(g) Peppers**	30%	30	131	30.50	5.86	0.0568	100	37	32.54	3.51	0.0449
	40%	26	80	28.66	4.41	0.0702	80	36	31.13	3.54	0.0528
	50%	23	91	26.66	3.63	0.0883	60	43	28.38	3.84	0.0725
	60%	15	66	24.57	2.68	0.1124	40	61	25.83	5.01	0.0972
**(h) Weatherstation**	30%	30	142	28.74	17.70	0.0751	100	31	30.69	11.08	0.0599
	40%	19	117	27.72	16.44	0.0844	80	37	29.55	13.28	0.0684
	50%	17	84	26.70	13.56	0.0949	60	34	28.00	15.97	0.0817
	60%	10	58	25.30	10.65	0.1115	40	61	25.94	20.06	0.1035

**Table 4:** Numerical comparison of CTY and our proposed method under 7 × 7 average blur and corrupted by salt-and-pepper noise from 30% to 60%.

**Images**	**Noise**	**CTY**					**Ours**				
	**level**	***μ***	**Itrs**	**PSNR**	**Time**	**ReE**	***μ***	**Itrs**	**PSNR**	**Time**	**ReE**
**(b) Satellite**	30%	21	72	29.45	2.71	0.1617	100	34	30.05	2.84	0.1510
	40%	18	57	28.66	2.23	0.1772	80	40	29.23	3.14	0.1660
	50%	14	48	27.69	1.92	0.1981	60	49	28.24	3.71	0.1860
	60%	9	41	26.74	1.84	0.2209	40	72	27.08	5.35	0.2126
**(c) House**	30%	20	105	33.61	3.90	0.0366	100	38	36.40	2.90	0.0265
	40%	12	75	32.63	2.93	0.0410	80	43	35.02	3.48	0.0311
	50%	10	65	31.69	2.66	0.0456	60	50	32.89	3.87	0.0397
	60%	7	60	30.18	2.51	0.0543	40	59	30.65	4.46	0.0515
**(d) Boat**	30%	27	138	28.54	5.43	0.0700	100	36	30.14	3.04	0.0582
	40%	21	104	27.40	4.23	0.0799	80	37	28.86	2.96	0.0675
	50%	17	82	26.19	3.10	0.0917	60	43	27.24	3.51	0.0813
	60%	13	65	24.87	2.50	0.1068	40	59	25.52	4.52	0.0991
**(e) Barbara**	30%	31	142	25.65	5.44	0.0976	100	32	27.16	2.56	0.0820
	40%	28	113	24.81	5.09	0.1075	80	39	26.02	3.37	0.0935
	50%	19	84	24.17	3.31	0.1157	60	44	24.94	3.53	0.1060
	60%	6	57	23.60	2.43	0.1237	40	61	23.97	4.85	0.1184
**(f) Einstein**	30%	22	117	31.80	4.54	0.0575	100	36	33.01	2.96	0.0500
	40%	15	88	31.11	3.45	0.0623	80	38	32.15	3.14	0.0552
	50%	14	74	30.00	2.98	0.0708	60	48	30.79	3.93	0.0646
	60%	12	64	28.36	2.40	0.0855	40	61	28.72	4.65	0.0820
**(g) Peppers**	30%	28	134	30.86	5.46	0.0545	100	38	32.71	3.03	0.0440
	40%	26	109	29.01	4.27	0.0674	80	39	31.30	3.21	0.0518
	50%	22	90	26.84	3.45	0.0865	60	44	28.63	3.71	0.0704
	60%	14	70	24.78	2.71	0.1097	40	60	26.04	4.66	0.0948
**(h) Weatherstation**	30%	25	117	28.87	21.53	0.0739	100	35	30.86	11.65	0.0588
	40%	23	98	27.84	17.94	0.0834	80	42	29.57	13.63	0.0682
	50%	15	73	26.70	13.32	0.0949	60	51	27.93	16.43	0.0824
	60%	10	60	25.26	10.55	0.1120	40	65	25.93	20.31	0.1037

**Fig 6:**
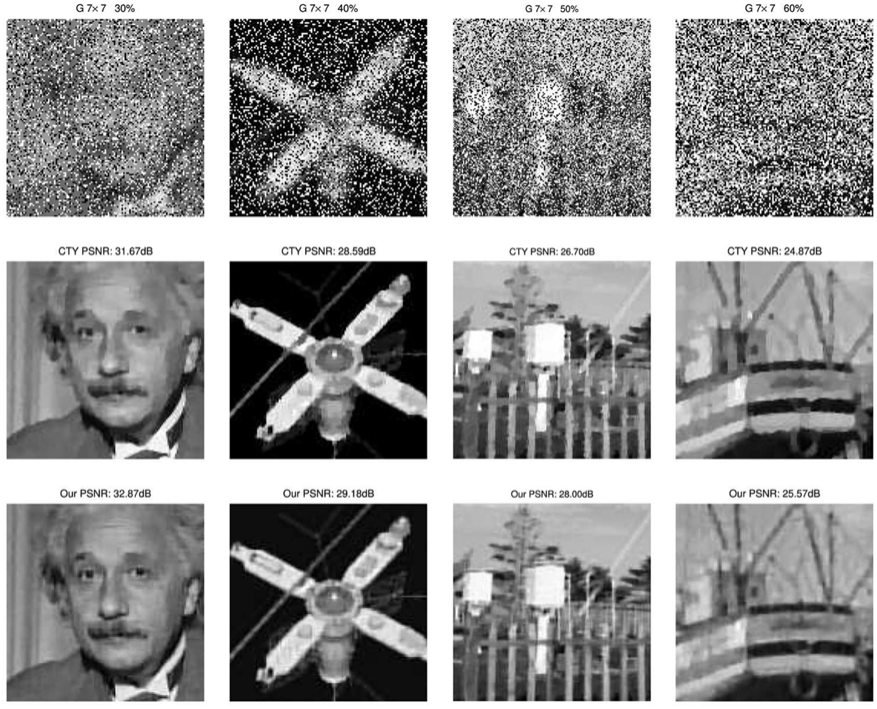
Several random examples of degraded and restored images of CTY and our method under Gaussian blur. Top row, zoom parts of blurred and noisy images under 7 × 7 Gaussian blur with standard deviation 5 and corrupted by salt-and-pepper noise. Second row, zoom parts of restored images by CTY respectively. Third row, zoom parts of restored images by our proposed method respectively. The PSNR results can be found clearly from Table 3.

**Fig 7:**
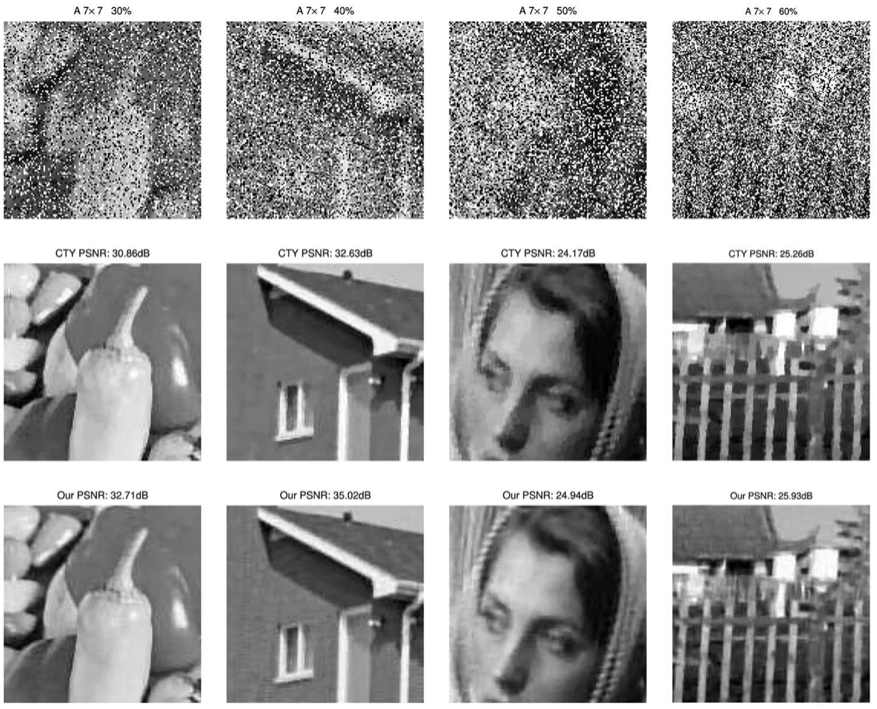
Several random examples of degraded and restored images of CTY and our method under average blur. Top row, zoom parts of blurred and noisy images under 7 × 7 average blur and corrupted by salt-and-pepper noise. Second row, zoom parts of restored images by CTY respectively. Third row, zoom parts of restored images by our proposed method respectively. The PSNR results can be found clearly from Table 4.

Finally, in Fig 6 and Fig 7, we display the zoom parts of the degraded images examples and the zoom parts of the restored test images by two methods respectively for Gaussian blur and average blur with noise level from 30% to 60%. We can easily see the visual improvement in the images by using our method. More specifically, from the third column in Fig 7, although the numerical result does not improve too much, the image visual quality of our proposed method is much better than CTY. This superiority is obvious for almost all the test images in our work.

### Comparison with other TV-based methods

For image restoration under Gaussian noise, there has been many literature for high order TV or other TV-based methods, such as Lysaker-Lundervold-Tai (LLT) model [36], Wu-Tai’s work [13], total generalized variation [39]. However, to our knowledge, the methods for image deblurring and denoising under impulse noise with these TV-based methods are still missing in the literature. More recently, Liu-Huang-Liu in [27] proposed a hybrid model for image deblurring and denoising under impulse noise, but their method might spend more time due to the combination.

In order to get a fair comparison of our method and other TV-based methods, we take the high-order TV model by changing the ℓ_2_-fidelity term in LLT to ℓ_1_ (“HOTV” for short) and the total generalized variation method by changing the ℓ_2_-fidelity term in [39] to ℓ_1_ (“TGV” for short) as examples. Other TV-based methods can be similarly treated. Moreover, we also impose the box constraint in these examples and solve them by ADMM to compare them with our method under Gaussian blur (i) and 30% to 40% impulse noise for diffident images. We only list the results for three images, “Cameraman”, “House” and “Weatherstation” for examples. The codes for HOTV and TGV are projected by ourselves based on ADMM. Furthermore, we think that the superiority of our method can be seen clearly from these tests.

**Fig 8:**
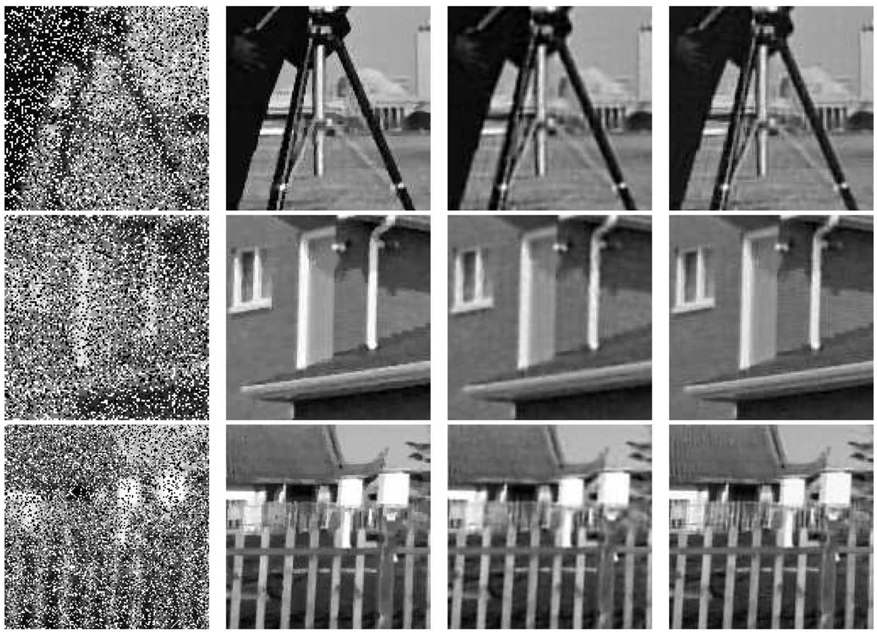
Degraded and restored images of TGV, HOTV and our method. Left column: zoom parts of blurred and noisy image. Second column: zoom parts of restored images by TGV. Third column: zoom parts of restored images by HOTV. Right column: zoom parts of restored images by proposed.

The numerical results are summarised in Table 5. The numerical improvement by our method is obvious from the table. Moreover, TGV usually performs better than HOTV but spends more time. Particularly, the time of each iteration by HOTV is less than other two methods ours and TGV. In Fig 8, we display the zoom parts of the degraded images examples and the zoom parts of the restored test images respectively for Gaussian blur with noise level 40% by three methods. We can see the visual improvement in the images by using our method. In addition, after test more experiments, we find that TGV preforms better than our method on the smooth region of the image, and our method performs better on the edges.

**Table 5:** Numerical comparison of our method, TGV and HOTV under Gaussian blur (i) and 30% to 40% impulse noise.

**Images**	**Noise**	**TGV**			**HOTV**			**Ours**		
	**level**	**Itrs**	**PSNR**	**Time**	**Itrs**	**PSNR**	**Time**	**Itrs**	**PSNR**	**Time**
**Cameraman**	30%	100	28.02	17.2	63	27.53	4.70	38	28.73	3.24
	40%	92	27.06	16.1	65	26.09	4.90	43	27.50	3.59
**House**	30%	90	35.31	15.8	55	35.08	4.09	37	36.20	2.96
	40%	95	33.42	16.5	61	32.66	4.65	42	34.99	3.45
**Weatherstation**	30%	75	29.88	42.1	63	29.51	19.45	31	30.69	11.08
	40%	80	28.47	45.4	67	28.04	20.45	37	29.55	13.28

**Remark 3**. In this work, we do not compare our method with other two stage methods such as Cai et. al [23] and Yan [24], because our method is a variational method by imposing a new regularization term. Our contribution is that we proposed a new convex model for handling the impulse noise without distinction of salt-and-pepper noise or random-value noise. We could easily apply our new regularization term in the former two stage methods and would consider it in future.

## Discussion and Conclusion

In this work, we study a new regularization model by applying TV with OGS in the classic ℓ_1_-TV model for the image deblurring under impulse noise. We provided the efficient algorithm CL1-OGS-ATV-ADM4 under the framework of general ADMM. In particular, an MM inner iteration is proposed to solve the subproblem instead of Shrinkage [20] in the classic ℓ_1_-TV model. The numerical results illustrate that our method outperforms CTY [26], GLN [8] and some other TV-based methods both in terms of the PSNR values, ReE, iterations or image visual quality. Our main contributions are firstly combining three existent parts together in one convex model for image deblurring under impulse noise without distinction of salt-and-pepper noise or random-value noise. Furthermore, we could easily apply our new regularization term in the popular two stage methods and would consider it in future.
